# Regulation of burstiness by network-driven activation

**DOI:** 10.1038/srep09714

**Published:** 2015-05-13

**Authors:** Guillermo García-Pérez, Marián Boguñá, M. Ángeles Serrano

**Affiliations:** 1 Departament de Física Fonamental, Universitat de Barcelona, Martí i Franquès 1, 08028 Barcelona, Spain

## Abstract

We prove that complex networks of interactions have the capacity to regulate and buffer unpredictable fluctuations in production events. We show that non-bursty network-driven activation dynamics can effectively regulate the level of burstiness in the production of nodes, which can be enhanced or reduced. Burstiness can be induced even when the endogenous inter-event time distribution of nodes' production is non-bursty. We find that hubs tend to be less susceptible to the networked regulatory effects than low degree nodes. Our results have important implications for the analysis and engineering of bursty activity in a range of systems, from communication networks to transcription and translation of genes into proteins in cells.

While human perception tends to appreciate regularity in the happening of events, evidence reveals that in many systems events cluster in bursts, *i.e.*, accumulations of a large number of rapidly occurring events during short time intervals separated by silent periods. Bursts have been experimentally and empirically observed in profusion and many models have been proposed to explain and generate them. For example, traces of human activity are to a great extent imprinted with burstiness. In particular, bursts and heavy-tails pervade the diversity of human communication channels, from written text^1^,^2^,^3^, to the frequency of letter^4^ and e-mail correspondence^5^,^6^, and cell phone calls^7^,^8^.

In a different context, key processes in single-cell biology, such as transcription and translation, are particularly prone to progress in bursts. For instance, mRNA synthesis proceeds in short but intense outbreaks beginning when coding genes transition from an inactive to an active state^9^. These bursts have been suggested to affect the expression of essential genes and even the switching and rhythms of cellular states^10^,^11^,^12^,^13^,^14^. This may raise questions about how cells are able to maintain function in the face of such unpredictable fluctuations, with the hypothesis that those bursts could be buffered by local or bulk degradation mechanisms^9^. It is interesting to explore whether large-scale system mechanisms, like the interconnection of genes in regulatory networks of interactions, have the capacity to produce similar buffering effects. It has already been observed experimentally that noise properties of small biological circuits depend on their architecture^15^ and computationally that the level of synchronized bursting in neuronal circuits is a direct function of global topology^16^. In the social sciences, the collective communication activity of a social online network has been reported to present long-term correlations not related to the distribution of inter-event times of single users^17^.

In this work, we show indeed that endogenous burstiness in the production of nodes can be both enhanced or reduced by stochastic network-driven non-bursty interactions, which activate/inactivate nodes dynamically. We use complex networks where nodes can present two states, active and inactive. When active, each node produces events according to a certain endogenous inter-event time probability distribution function 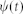. The activation dynamics is governed by a process propagating in the network akin to the standard Susceptible-Infected-Susceptible epidemic spreading model^18^, so that active nodes inactivate at rate 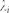 and inactive ones become active upon contact with active neighbors at rate 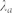 per active contact. In all cases, these processes are assumed to follow Poisson statistics. As a result, we observe that non-bursty network-driven activation dynamics can effectively regulate the level of burstiness in the production of nodes even when their endogenous production pattern is non-bursty. We find that hubs tend to be less susceptible to the networked regulatory effects than low degree nodes. This is a general result for systems where the parameters of dynamics, production, and topology are independent of each other. Note that here we are reversing the topical question about the macroscopic collective effects of burstiness on dynamical processes running on complex networks. The bursty activity of nodes, both in models of opinion formation^19^,^20^ and epidemic spreading^21^,^22^,^23^, has been observed to induce a slowing down of the progression of the dynamical process. Here, we focus instead on the regulatory effects of networked dynamical interactions on the endogenous bursty activity of individual nodes.

The problem can be posed in terms of a single node *l* that changes state following Poisson processes with an instantaneous activation rate 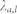 —which encodes the network effect on the alternation of activation periods of nodes—and inactivation rate 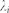. The activation rate 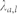 of node *l* is a stochastic process that depends on the number of active neighbors of *l* at time *t*. Nevertheless, when node degrees are the relevant feature of the network topology, once at the steady state, the instantaneous activation rate of a node of degree 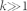 can be replaced by an effective activation rate 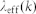 depending on the degree distribution *P*(*k*) and degree-degree correlations *P*(*k*′|*k*)^24^, with a temporal average value given by
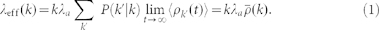
Here *k* is the node degree and *ρ_k_*_′_(*t*) is the fraction of active nodes (prevalence) of degree *k*′, so that 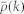 is a temporal average of a degree-weighted prevalence depending on degree-degree correlations. In uncorrelated networks, *P*(*k*′|*k*) = *k*′*P*(*k*′)/⟨*k*⟩ and the degree weighted prevalence is degree-independent, so that 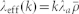. This temporal average fluctuates around a constant value in the stationary state of the endemic phase (see Supplementary Information).

Such a node changes state following Poisson processes with rates 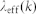 for activation and 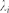 for inactivation and, when active, produces events according to a general distribution 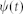 with average ⟨*t_p_*⟩. For any 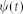, the effective inter-event time probability density function of production events *φ*(*t*,*k*) can be analytically calculated as
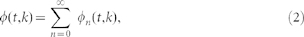
where *φ_n_*(*t*,*k*) is the probability that the time between two consecutive production events is within the interval (*t*, *t* + *dt*) after exactly *n* inactive periods. These partial densities can be calculated in terms of convolution integrals and, in the Laplace space, the sum in Eq. (2) can be carried out analytically (see Methods for details), yielding the following expression for 
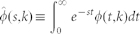

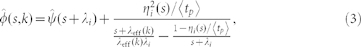
where 
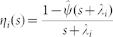
. We have used that the probability density for the interval between the activation time and the first production event is given by 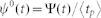, where 
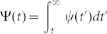
 is the survival probability function. The rationale for this choice is that the activation event can be regarded as taking place between two consecutive production events. Since we do not have any other information, we assume that the time interval between those two events is greater than the time elapsed between the activation event and the first production. As a consequence, its probability density must be proportional to 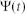, and the denominator simply normalises the distribution. Hence, effective inter-event times of production events are ruled by the interplay of three key factors: the endogenous production statistics 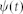, the first production event statistics 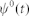, and the activation dynamics through the effective activation rate 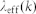, that for uncorrelated networks depends linearly on degree.

To measure the burstiness of production, we use the burstiness coefficient 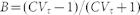 as defined in Ref. 25, where 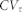 is the coefficient of variation 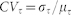 and 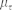 and 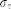 are the mean and standard deviation of the time between consecutive production events. With this definition, *B* = 1 corresponds to a strongly bursty production, *B* = 0 to a neutral one following a Poisson statistics, and *B* = −1 to a periodic signal. As an example, we now particularize to the case of a Weibull distribution for production events
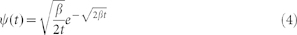
with scale parameter (2*β*)^−1^ adjusting the spread of the distribution, and shape parameter 1/2 implying a heterogeneous non-Poisonian distribution such that all nodes have an endogenous production of events that clusterize in time. Weibull distributions –the stretched exponential function is the complementary cumulative distribution function of the Weibull– are used extensively to model heterogeneous distribution of events but with finite moments^26^. In our particular case, it has the advantage of being analytically solvable. The endogenous burstiness for a node continuously producing according to Eq. (4) is 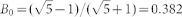, independent of *β*.

The effective value of the burstiness of a node with interrupted production due to the network-driven activation/inactivation dynamics depends on its degree, *B*(*k*). To compute it, we need to calculate the degree-dependent average and standard deviation of production inter-event times, which can be easily evaluated through simple derivatives of Eq. (3) evaluated at *s* = 0 (see Methods). The degree dependent coefficient of variation reads
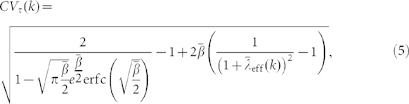
where we have redefined 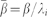 and 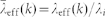. As a validation, we display in Fig. 1a the analytical result for *B* based on Eq. (5) along with the simulation of a single node whose state changes following Poisson processes with rates 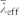 for activation and 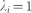 for inactivation, and that intrinsically produces events according to Eq. (4) with rate 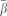. The agreement between the analytical surface and the simulation points is excellent. Both results prove that the level of endogenous burstiness for continuous production *B*_0_ = 0.382 can be both increased and decreased, as shown also in the projection of the analytical surface on the 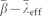 plane in Fig. 1b. The effective burstiness ranges always between zero burstiness (exponential inter-event time distribution) in the limit 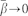 and the maximum of 1 when 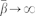. For high values of 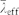, *B* is dominated by the interplay of the endogenous production statistics 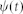 and the first production event statistics 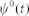. The range of 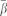 values associated to effective burstiness around *B*_0_ widens with increasing 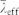 and the endogenous production level is recovered only when both 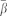 and 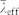 are increased simultaneously. In contrast, the effective burstiness raises for low values of 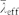 due to the effect of increased inactivation periods, such that for each 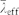 the level of effective burstiness increases with 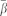. These results are qualitatively the same for any distribution 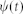.

In uncorrelated networks, the proportional dependence of 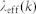 with degree Eq. (1) implies that the effective burstiness is a decreasing function of *k*. To confirm this, we measured *B*(*k*) from simulations on a network with *N* = 10^4^ nodes (details in Supplementary Information). Our results are valid for different degree distributions, but we used a scale-free network with characteristic exponent 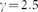. In Fig. 2a, we show simulation results of *B*(*k*) for different values of 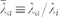. For all activation rates, the effective burstiness decreases with degree. For low degree nodes, 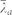 determines the duration of inactive periods. Low values of 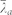 (but above the minimum required to sustain the activity) enable low degree nodes remaining a long time in the inactive state, which raises their effective burstiness well above *B*_0_, while for high values of 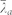 they behave as high degree nodes approaching a minimum value of *B*(*k*) independent of *k*, a basal level below *B*_0_. For high degree nodes, *B*(*k*) is noticeably below *B*_0_. This is due to the fact that hubs do not remain in the inactive state much time since they are usually connected to many active nodes that constantly reactivate them. This fact, combined with variable values of 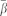, can produce levels of effective burstiness different from the endogenous one (Fig. 2b). In the limit of very small 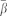, it is indeed possible to bring effective burstiness to zero since the effective production becomes a Poisson process.

Fixing 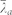, the production shape parameter 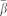 can be varied to regulate the effective level of burstiness, Fig. 2b, with similar qualitative behavior. In both cases, the disparity of effective *B*(*k*) values reaches a maximum at some intermediate parameter levels. However, 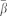 does not have any limitation to be increased (in our computational experiments it is varied over four orders of magnitude) so that high-degree nodes can increase their effective burstiness above the endogenous level. Taken together, our results indicate that the bursty production of nodes can be regulated by network-driven stochastic activation dynamics. When all nodes produce equally, the originally uniform endogenous burstiness is split in a range of values anti-correlated with degree. More in general, low-degree nodes tend to be more susceptible to the networked regulatory effects than high-degree nodes, presenting a broader variability of the effective burstiness as a function of the parameters of both the dynamical process and the production function.

Finally, we also prove that network-driven activation can induce burstiness even when the endogenous production of nodes is not bursty. We let nodes in the active state to produce events with an exponential inter-event time probability distribution function of rate *α*, 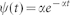, which has *B*_0_ = 0. Again, calculating the first two moments of Eq. (3) (see Methods), we obtain
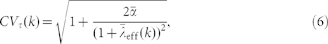
where 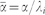. This analytical expression matches almost perfectly with numerical simulations of a single node whose state changes following Poisson processes with rates 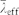 for activation and 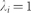 for inactivation, and that intrinsically produces events according to a exponential inter-event time probability distribution with rate 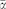, Fig. 3a. For an exponential distribution, 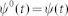 so that 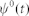 has no role, as expected for a Poisson process. Hence, the effective burstiness can only be equal or above the endogenous value *B*_0_ = 0. This induced burstiness is explained as a consequence of the fact that the node may remain in the inactive state for a period of time considerably longer than the average endogenous inter-event time ⟨*t_p_*⟩ = 1/*α*^27^.

We also simulated exponential production in the same scale-free network with *N* = 10^4^ nodes and characteristic exponent 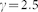 used in the study with the Weibull distribution. We used different values of 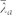 with 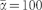, Fig. 3b, and different values of 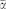 over four orders of magnitude with 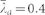, Fig. 3c. As for bursty production, we find again that *B*(*k*) is always a decreasing function of *k* (but always above *B*_0_ = 0). Due to their sustained activity, hubs are less susceptible to display induced burstiness production. In contrast, nodes with low degree stay in the inactive state for longer periods and are more prone to present a raised effective burstiness. Qualitatively, both parameters 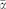 and 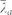 can be modified to induce burstiness, affecting all nodes more efficiently for higher values of 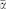 and lower values of 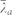. Again, the disparity of induced burstiness values reaches a maximum at some intermediate parameter levels.

Different dynamical processes occurring on a network can interplay in unexpected ways. We have seen here that the time series of production events of interacting nodes are affected by network-driven activation/inactivation dynamics. In a broad range of parameters, hubs in a network tend to decrease burstiness in their endogenous pattern of production, hence helping to maintain a stable bulk level, at the expenses of low degree nodes being more pliable to the regulatory effect of the network that tends to amplify their fluctuations. At the same time, lower degree nodes are more prone to burstiness induction. In general, hubs tend to be less susceptible to the networked regulatory effects than low degree nodes. These results indicate that a heterogenous network structure protects the functioning of some nodes, the hubs, making low degree nodes better targets for engineering actions to produce local modifications of production without critically affecting the behavior of the whole system. Taken together, our findings suggest that heterogeneous network interconnectivity may be a strategy in itself developed to protect complex systems against unpredictable functional fluctuations. However, further research should be conducted to determine the effects of different activation/inactivation dynamics on node's burstiness.

## Methods

### Calculation of the probability density function *φ*(*t,k*) in the Laplace space

The result in Eq. (1) reduces the study of the system to that of a single node that activates with rate 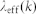, deactivates with rate 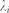 and produces in the active state with probability densities 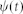 and 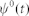. Let 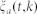 and 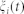 denote the probability densities for activation and deactivation, and let Ξ*_i_*(*t*) and 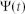 stand for the survival probabilities of 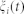 and 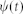. We now compute the probability *φ*(*t,k*)*dt* that two consecutive production events take place in the interval (*t, t + dt*) taking into account the fact that the state of the node can flip any number of times between the two events (as long as it is in the active state during both productions). It is easy to see that if it does not flip to the inactive state during the process, *φ*(*t,k*)*dt* is given by 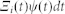 (the probability that it does not deactivate in a time less than *t* and that it produces in the given interval). Similarly, we can compute the probability that it deactivates only once between both productions; however, in this case we have to integrate for all possible deactivation and activation times (let us call them *t*_1_ and *t*_2_, respectively), yielding 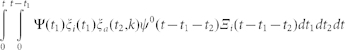. Following the same procedure, we can write the total probability density as the series
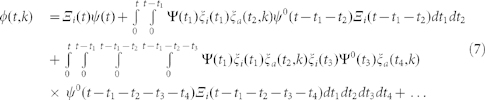
We do not need to solve the series, since we are only interested in the first two moments of the distribution to obtain *B*(*k*). To this end, we make use of the Laplace transform formalism, which is particularly convenient for two reasons. On the one hand, the *n*-th moment of the distribution *φ*(*t,k*) can be obtained as the *n*-th derivative of its transform 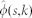, 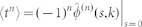. On the other hand, the transform of a convolution is 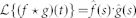. Since any term in Eq. (7) is a chain of convolutions, it is not hard to write down 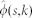,
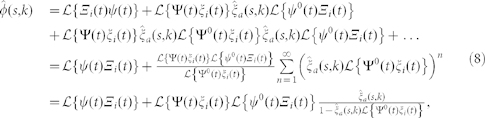
where in the last step we have used that 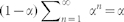. We can write the latter result in terms of 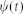 only by plugging the expressions for 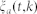 and 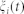 into it and using
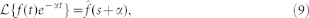

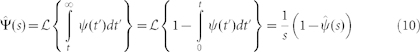
and
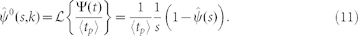
The final expression for 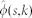 reads
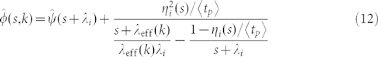
with 
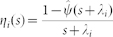
, which is Eq. (3).

### Derivation of the coefficient of variation for a Weibull density function

Plugging the Laplace transform of Eq. (4)
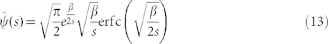
into Eq. (12) yields a rather cumbersome expression,
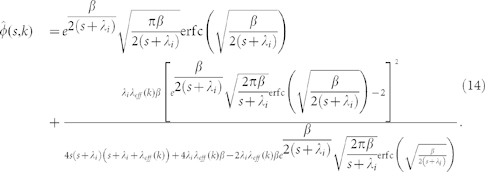
We can now obtain the first and second moments of *φ*(*t,k*) deriving the latter equation and therefore write the expressions for the mean 
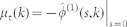
 and the standard deviation 
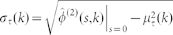
 in terms of the normalized parameters 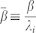 and 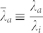:
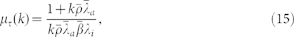

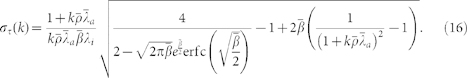
The quotient of both expressions gives Eq. (5).

### Derivation of the coefficient of variation for an exponential density function

The Laplace transform for an exponential 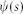 is given by
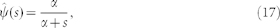
with which Eq. (12) becomes
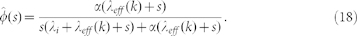
Evaluating its first and second derivatives at *s* = 0 and using normalized parameters 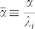 and 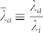 gives
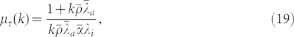
and
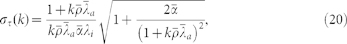
from which Eq. (6) can be immediately obtained.

## Supplementary Material

Supplementary Information Supplementary Information

## Figures and Tables

**Figure 1:**
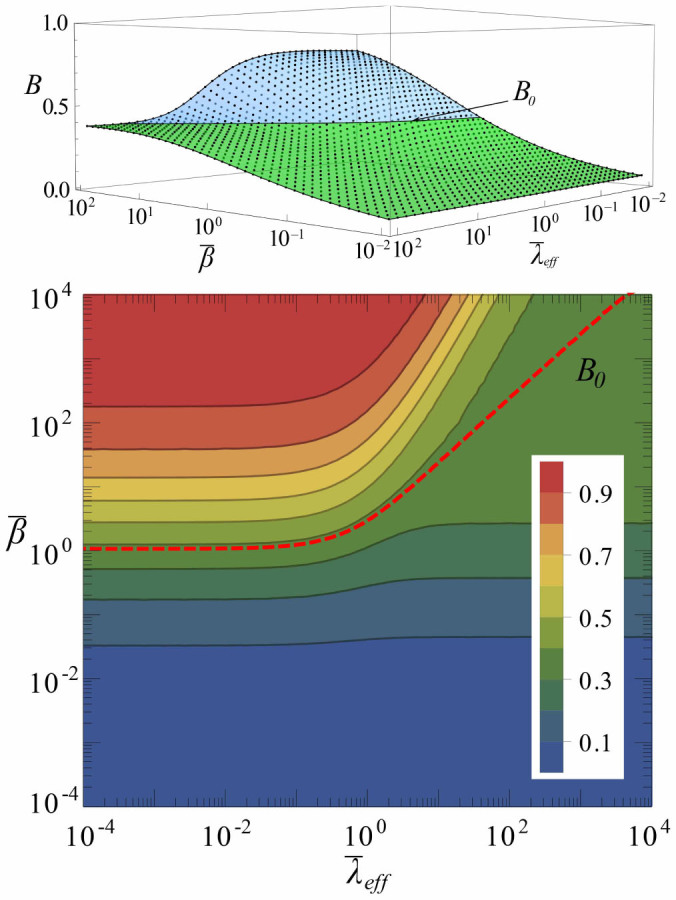
Regulation of burstiness. (a). Comparison between simulation data (dots) and the analytical calculation (solid surface and line) of the effective burstiness 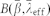, *B* > *B*_0_ = 0.382 in the blue region and *B* > *B*_0_ = 0.382 in the green one. (b). Projection of burstiness surface in (a) in the 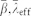 plane. Colors in the contour plot code for different values of *B*.

**Figure 2:**
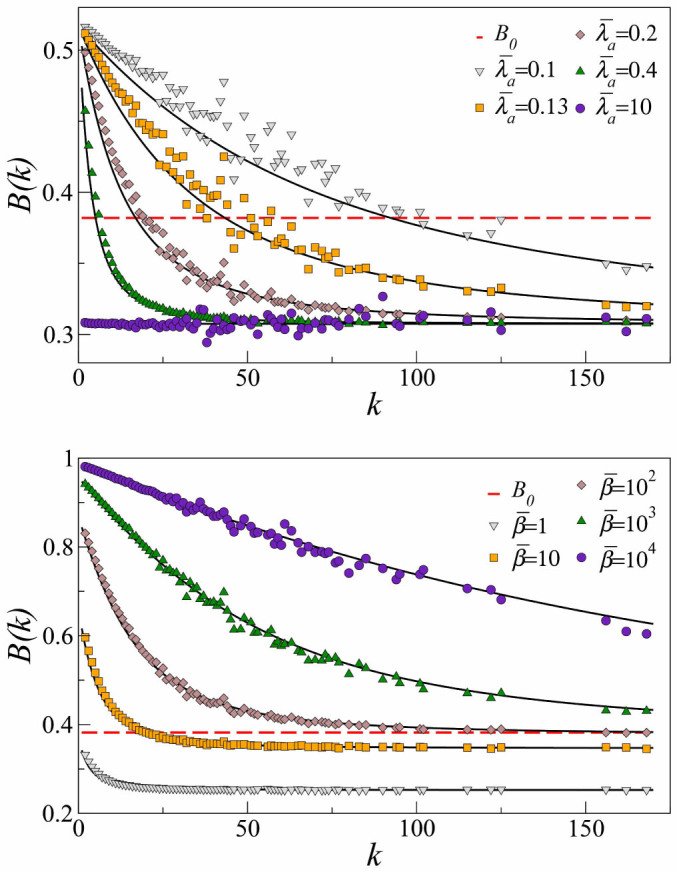
Network-driven regulation of burstiness. (a). *B*(*k*) for different values of the activation rate 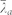 and fixed 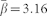, chosen so that a wide heterogeneity of behaviors can be observed and *B*_0_ lies about the middle of the interval in which *B*(*k*) can be varied. (b). *B*(*k*) for fixed 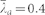 and different values of the production scale parameter 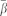.

**Figure 3:**
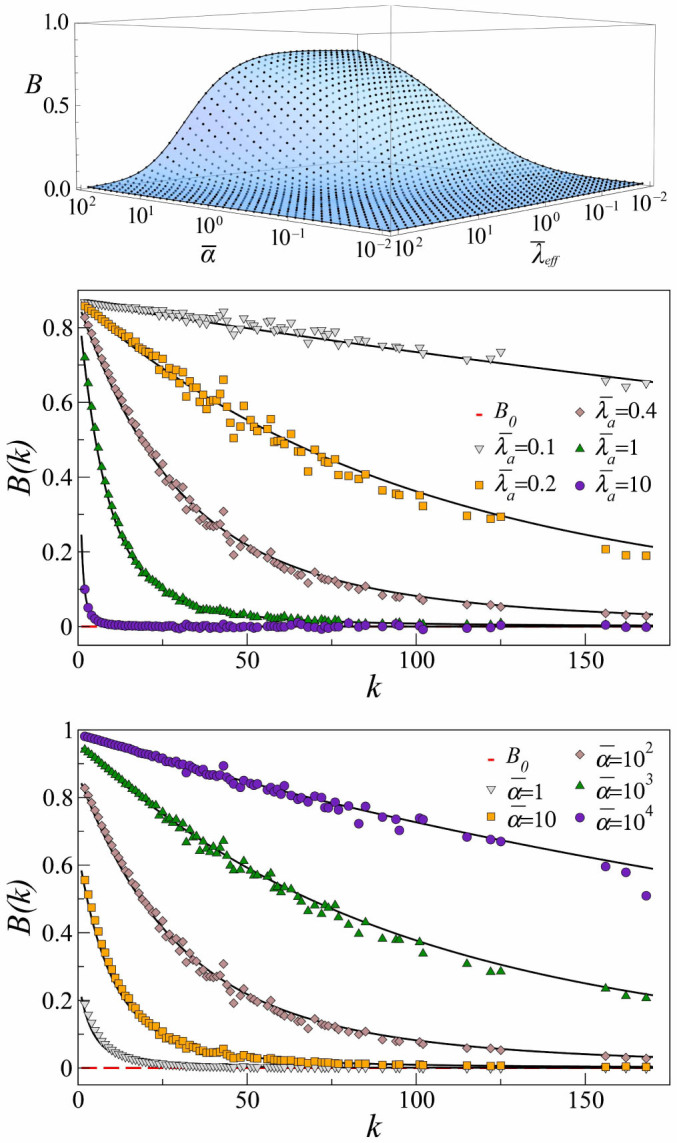
Network-driven induction of burstiness. In all graphs, solid surface and lines correspond to analytical results and dots to simulations. (a) Comparison between simulation data and the analytical calculation of the effective burstiness 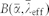, *B* > *B*_0_ = 0. (b). *B*(*k*) for fixed 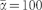 and different values of the activation rate 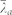. (c). *B*(*k*) for fixed 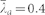 and different values of the production rate 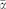.
