# Supplementary material for: Regulation of burstiness by network-driven activation
Source: Sci Rep. 2015 May 13;5:9714. doi: 10.1038/srep09714 (PMC4429350; doi:10.1038/srep09714)
Supplement: Supplementary Information [file srep09714-s1.pdf]

Supplementary information for "Regulation of burstiness by network-driven activation"

Guillermo García-Pérez,[1] Marián Boguñá,[1] and M. Ángeles Serrano[1]
[1]*Departament de Física Fonamental, Universitat de Barcelona, Martí i Franquès 1, 08028 Barcelona, Spain*

Contents

I. Dynamics and notation 1

II. Limiting cases and scope of the results 2

III. Simulation details 3

IV. Effective burstiness in different heterogeneous topologies 4

References 5

I. DYNAMICS AND NOTATION

The system is composed of a set of elements interconnected forming a scale-free undirected random network. Every node can present two states, active and inactive, and the dynamics that governs their state is similar to the standard SIS model. Nodes flip from one state to the other following a Poisson process with probability densities $\xi_a(t)$ for activation and $\xi_i(t)$ for deactivation. In particular, a node l flips from the active to the inactive state at a constant rate λ_i, while its activation rate is proportional to the number of active nodes to which it is connected, $\lambda_{a,l}(t) = \lambda_a \sum_m a_{lm} n_m(t)$. In the latter sum, $n_m(t) = 1$ if node m is active at time t and $n_m(t) = 0$ otherwise; similarly, $a_{lm} = 1$ if and only if nodes l and m are connected.

We now show that $\lambda_{a,l}(t)$ is, after the system has reached the steady-state, approximately time-independent and proportional to the degree of the node k_l [1]; we will refer to it as $\lambda_{eff}(k)$. To derive its expression, we first apply a coarse-graining approximation by assuming that all nodes with the same degree k are equivalent and statistically independent. Under this approximation, the state of the system is characterized by the fraction of nodes in the active state in every degree class,

$$\rho_k(t) = \frac{\sum\limits_{i|k_i=k} n_i(t)}{N_k}, \tag{S1}$$

where N_k stands for the number of nodes with degree k in the network. Since we are assuming all nodes with the same degree to be statistically independent, $\rho_k(t)$ yields the probability that a randomly chosen node with degree k is active. Hence, the probability that a randomly chosen link emerging from a node with degree k reaches an active node can be computed as $\sum_{k'} P(k'|k)\rho_{k'}(t)$, i.e., the probability that the link reaches a node with degree k', $P(k'|k)$, and that such node is active, $\rho_{k'}(t)$, summed for all k'. In the case of uncorrelated random networks, $P(k'|k) = k' \frac{P(k')}{\langle k \rangle}$, so the probability for a link to connect to an active node becomes degree independent, $\rho(t) \equiv \sum_{k'} \rho_{k'}(t) k' \frac{P(k')}{\langle k \rangle}$. Moreover, in the steady-state it takes an almost constant value $\bar{\rho} \equiv \left\langle \sum_{k'} \rho_{k'}(t) k' \frac{P(k')}{\langle k \rangle} \right\rangle$ (see Fig. S1), which allows us to compute the average number of active neighbors of a node with degree k as $k\bar{\rho}$. Hence,

$$\lambda_{eff}(k) = \lambda_a k \bar{\rho} = \lambda_a k \left\langle \sum_{k'} \rho_{k'}(t) k' \frac{P(k')}{\langle k \rangle} \right\rangle. \tag{S2}$$

Notice that λ_{eff} is a function of degree for a wide class of activation-deactivation dynamics (not necessarily SIS-like), as long as the system has absorbing states. In a more general situation, the activation rate of a node i is given by an

arbitrary function f of the state of its neighbours, i.e.

$$\lambda(i,t) = f\left(\sum_j a_{ij} n_j(t)\right). \tag{S3}$$

Coarse-graining by degree classes yields

$$\lambda(k,t) = f\left(k \sum_{k'} P\left(k|k'\right) \rho_{k'}(t)\right). \tag{S4}$$

The effective activation rate is the temporal average of the latter quantity,

$$\lambda_{eff}(k) = \left\langle f\left(k \sum_{k'} P\left(k'|k\right) \rho_{k'}(t)\right)\right\rangle. \tag{S5}$$

As a consequence, the approach followed in this work can be applied to the case of correlated network topologies and activation-inactivation dynamics with stationary states as well (see Figs. S2 and S3). We have chosen uncorrelated networks and SIS-like dynamics for the sake of simplicity.

In addition to the SIS-like dynamics, we let nodes produce while they are in the active state with a probability density $\psi(t)$, which need not be exponential. It is therefore important to note that, if $\psi(t)dt$ gives the probability for the time interval between two consecutive production events to fall into $(t, t+dt)$ (for a node that is active along all the process), the probability density for the interval between the activation time and the first production event $\psi^0(t)$ is, in general, given by

$$\psi^0(t) = \frac{\Psi(t)}{\langle t_p \rangle}, \tag{S6}$$

where $\langle t_p \rangle = \int\limits_0^\infty t\psi(t)dt$ is the average production interval and $\Psi(t) = \int\limits_t^\infty \psi(t')dt'$ is the survival probability distribution (the probability that the interval is greater than t). We will use the same notation for the activation and deactivation probabilities as well: $\Xi_{a/i}(t)$ will refer to the survival probability of $\xi_{a/i}(t)$. To justify Eq. (S6), let us consider a sequence of $N \to \infty$ events $t_i, i = 1, \ldots, N$ distributed with probability density $\psi(t)$ (so the whole process takes a time $T = \sum_{i=1}^N t_i$) and calculate $\psi^0(t)dt$, i.e., the probability that an activation event falls at a distance between t and $t + dt$ from the next production event. Assuming that the activation event is uniformly distributed along the interval $(0, T)$, the probability for it to fall in any interval of length L is simply $\frac{L}{T}$. In our case, $L = N_{t_i>t}dt$, where $N_{t_i>t}$ is the number of intervals greater than t, since there is an interval of length dt placed at distance t from the next production event in every production interval greater than t. Therefore,

$$\psi^0(t)dt = \lim_{N\to\infty} \frac{N_{t_i>t}dt}{T} = \lim_{N\to\infty} \frac{\frac{N_{t_i>t}}{N}}{\frac{\sum\limits_{i=1}^N t_i}{N}} dt = \frac{\Psi(t)}{\langle t_p \rangle} dt. \tag{S7}$$

II. LIMITING CASES AND SCOPE OF THE RESULTS

Let us focus on the case of a single node that produces with an arbitrary $\psi(t)$ and consider several limiting cases. In order to define a timescale, we set $\beta \equiv \langle t_p \rangle^{-1} = 1$ throughout all this section without loss of generality (so the ratios λ_i/β and λ_{eff}/β to which we will refer become simply λ_i and λ_{eff}).

In the limit of $\lambda_i \to 0$, the fraction of production intervals interrupted by a deactivation goes to zero, so the resulting probability density function $\phi(t) \to \psi(t)$. This result can also be derived by taking the limit of Eq. (3) in the paper as $\lambda_i \to 0$, since $\hat{\phi}(s) \to \hat{\psi}(s)$. Notice, however, that this limit is slightly different to the limit $\beta/\lambda_i \to \infty$ and fixed λ_{eff}/λ_i (which gives $B(k) \to 1$); the reason is that, in the present case, when taking the limit $\lambda_i/\beta \to 0$ for a fixed λ_{eff}/β, the ratio β/λ_{eff} obviously remains constant, i.e. the interrupted intervals become less frequent but not longer than uninterrupted ones. On the other hand, taking the limit $\beta/\lambda_i \to \infty$ for a fixed λ_{eff}/λ_i gives $\beta/\lambda_{eff} \to \infty$. Therefore, even though interrupted intervals become less frequent, they become much longer than uninterrupted intervals as well, hence yielding a bursty behavior. That is why, when working with the normalized

parameters $\bar{\beta} = \beta/\lambda_i$ and $\bar{\lambda}_{eff} = \lambda_{eff}/\lambda_i$, $\bar{\lambda}_{eff}$ must be increased too in order to recover the original burstiness coefficient B_0 (as shown in Fig. 1 in the paper).

For intermediate values of the deactivation rate $0 < \lambda_i < 1$, the fraction of production events interrupted by a deactivation may be significant; there is, on average, one production event per unit time during the active state, while there are λ_i interruptions per unit time. Therefore, approximately one out of every λ_i^{-1} production intervals is interrupted. In addition, the average time the node remains in the inactive state is $\lambda_{eff}(k)^{-1}$, so if $\lambda_{eff}(k) \ll 1$, λ_i^{-1} production events buffer during the active state according to $\psi(t)$ and are followed by a long period of inactivity. Hence, the resulting distribution becomes burstier than the original.

In the opposite case ($\lambda_i \to \infty$), the production process becomes Poisson regardless of $\psi(t)$; since $\langle t_p^0 \rangle \gg \langle t_i \rangle$, the probability that the node produces during an active period (which we will denote by ν) is very small, and we can regard the process as a succession of active and inactive states of average length $\langle t_{a+i} \rangle = \frac{1}{\lambda_{eff}(k)} + \frac{1}{\lambda_i}$, each of which with an independent production probability ν. Under this approximation, we can easily compute the probability that two consecutive production events take place in a time interval T greater than t as the probability for the node not to produce in any of $\frac{t}{\langle t_{a+i} \rangle}$ intervals,

$$\text{Prob}\{T > t\} = (1-\nu)^{\frac{t}{\langle t_{a+i} \rangle}} = e^{\frac{t}{\langle t_{a+i} \rangle}\ln(1-\nu)} \approx e^{-\frac{\nu}{\langle t_{a+i} \rangle}t} = e^{-\frac{\lambda_i \lambda_{eff}(k)\nu}{\lambda_i + \lambda_{eff}(k)}t}. \tag{S8}$$

Furthermore, ν can be estimated as well for any $\psi(t)$. Its exact expression is given by

$$\nu = \int_0^\infty \psi^0(t)\Xi_i(t)dt = \int_0^\infty \Psi(t)e^{-\lambda_i t}dt. \tag{S9}$$

Since we are considering the limit of $\langle t_p^0 \rangle \gg \langle t_i \rangle$, the probability that a production interval is greater than $\langle t_i \rangle$ is approximately one, so $\Psi(t) \sim 1$ in the range of small values of t in which the exponential term in the integrand is not close to zero. Hence, we can approximate Eq. (S9) as

$$\nu \sim \int_0^\infty e^{-\lambda_i t}dt = \frac{1}{\lambda_i}. \tag{S10}$$

We therefore see that the production process becomes exponentially distributed with mean

$$\mu_\tau(k) = \frac{\lambda_i + \lambda_{eff}(k)}{\lambda_{eff}(k)}, \tag{S11}$$

which is in accordance with Eqs. (15,19) in the paper. This result can also be obtained by taking the limit of $\hat{\phi}(s)$ as $\lambda_i \to \infty$ assuming

$$\lim_{\lambda_i\to\infty} \hat{\psi}(s+\lambda_i) = \lim_{s\to\infty} \hat{\psi}(s) = \lim_{s\to\infty} \int_0^\infty e^{-st}\psi(t)dt = 0 \tag{S12}$$

and

$$\lim_{\lambda_i\to\infty} \Psi(s+\lambda_i) = \lim_{\lambda_i\to\infty} \frac{1}{s+\lambda_i}\left(1-\psi(s+\lambda_i)\right) = \lim_{\lambda_i\to\infty} \frac{1}{s+\lambda_i}. \tag{S13}$$

From all the stated above, we can conclude that the network dynamics can be used to regulate the production of nodes between three regimes of special interest: the endogenous production behavior, a bursty process and a Poisson process (see Table S1). Furthermore, these results are completely general and independent of the functional form of $\psi(t)$ (as long as Eq. (S12) holds).

III. SIMULATION DETAILS

The IAI dynamics is simulated using the continuous-time Gillespie algorithm. In addition, at every deactivation event the deactivated node's production is updated by generating random intervals with distributions $\psi^0(t)$ for the first one and $\psi(t)$ for the rest of them. The initial condition for all simulations is $n_i(0) = 1$, $\forall i$ (all nodes active),

Parameters range	$\phi(t)$	B
$\lambda_i/\beta \to 0$ and fixed $\lambda_{eff}(k)/\beta$	$\psi(t)$	B_0
$0 < \lambda_i/\beta < 1, \lambda_{eff}(k)/\beta \ll 1$	Bursty distribution	$> B_0$
$\lambda_i/\beta \to \infty$	$\frac{\lambda_{eff}(k)\beta}{\lambda_i+\lambda_{eff}(k)} e^{-\frac{\lambda_{eff}(k)\beta}{\lambda_i+\lambda_{eff}(k)}t}$	0

TABLE S1: Different behaviors exhibited by a node following the dynamics presented in the paper.

but we only measure nodes' B for simulation times $t > 100$, since the system reaches a stationary state before that value (see Fig. S1). We stop simulations when all nodes have produced at least 10^4 times, so enough statistics are guaranteed.

In Figs. 2 and 3 in the paper, the simulation parameters have been chosen in such a way that a wide heterogeneity of behaviours is exhibited; in Figs. 2a and 3b, every simulation corresponds to a different value of $\bar{\lambda}_a$ between $\bar{\lambda}_a = 0.1$ and $\bar{\lambda}_a = 10$, so $\bar{\rho}$ takes values approximately between $\bar{\rho} \approx 0.1$ and $\bar{\rho} \approx 1$. Since the degree of nodes is between $k = 1$ and $k \approx 170$, $\bar{\lambda}_{eff}(k)$ is approximately in the intervals $(0.01, 1.7)$ for $\bar{\lambda}_a = 0.1$ and $(10, 1700)$ for $\bar{\lambda}_a = 10$. From Fig. 1 it can be seen that for $\bar{\beta} \approx 3$, B_0 lies about the middle of the interval in which B can be varied; we have set $\bar{\beta} = 3.16$. A similar criterion has been applied when setting $\bar{\alpha} = 100$. Likewise, in Figs. 2b and 3c $\bar{\lambda}_a = 0.4$ is fixed, so $\bar{\rho} \approx 0.54$ and hence $\bar{\lambda}_{eff}(k) \in (0.2, 37)$. Given that $\bar{\alpha}$ and $\bar{\beta}$ are varied over 4 orders of magnitude, a wide range of B values is obtained.

IV. EFFECTIVE BURSTINESS IN DIFFERENT HETEROGENEOUS TOPOLOGIES

In this section we present some results not included in the paper. In Fig. S1 we show the quantity $\rho(t) = \sum_{k'} \rho_{k'}(t) k' \frac{P(k')}{\langle k \rangle}$ introduced in Section I. As expected, it fluctuates around a constant value that can be easily measured from simulations.

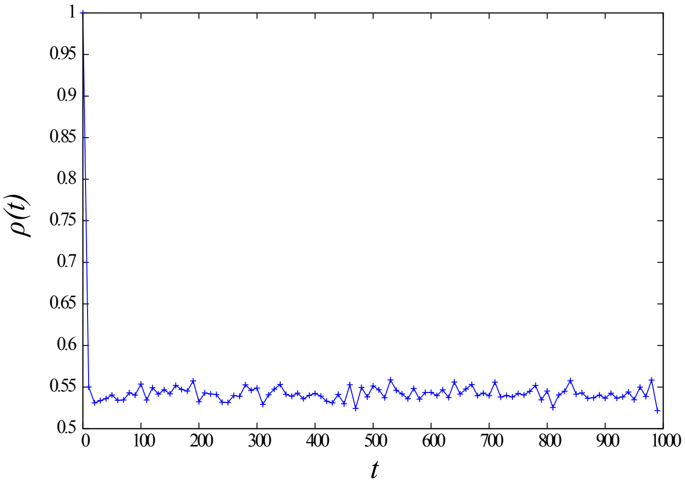

FIG. S1: Probability for a link to be active as a function of time $\rho(t)$.

To see how the network topology affects the induction of burstiness, we have measured $B(k)$ for different networks. In Fig. S2 we show the results for four SF networks with $N = 10^3$, $\langle k \rangle \approx 4$ and $\bar{\lambda}_a = 0.4$, but different exponents. In Fig. S3, the results correspond to four SF random graphs with the same degree distribution but different assortativities. Our results show a weak dependence on degree heterogeneity; indeed, the higher the heterogeneity, the faster the decay of $B(k)$ with k, although the effect is not really significant. Similarly, degree correlations slightly affect the burstiness of high degree nodes, since it is greater for disassortative networks. Yet, assortativity does not play an important role at burstiness induction either.

Since the burstiness coefficient $B(k)$ alone does not provide any details about the distribution other than the ratio between σ_τ and μ_τ, we have measured the cumulative inter-event time distribution function for the burstiest node in the network, as shown in Fig. S4 (left). We have also measured the burstiness of the same distribution by computing the normalized conditional average interval $\langle \tau | t \rangle / \langle \tau \rangle$ as a function of $t/\langle \tau \rangle$ (see Fig. S4, right). For a Poisson process, the conditional average interval $\langle \tau | t \rangle$ is independent of t and equal to $\langle \tau \rangle$, while for a regular-like distribution, this

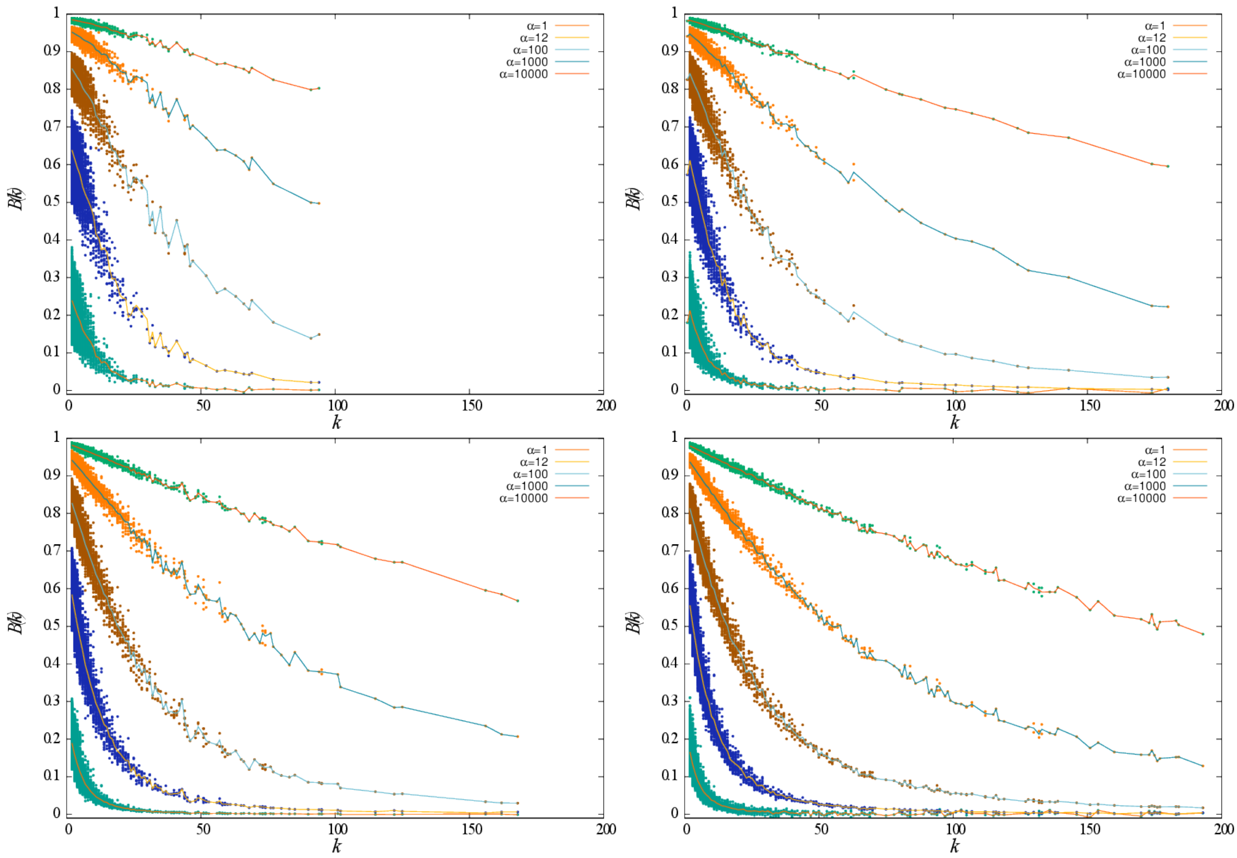

FIG. S2: $B(k)$ for SF graphs with different exponents γ. Top-left: $\gamma = 3$. Top-right: $\gamma = 2.75$. Bottom-left: $\gamma = 2.5$. Bottom-right: $\gamma = 2.25$.

should be a decreasing function (the more time elapsed since the last event, the less time is expected until the next one). In our case, we observe exactly the opposite situation, which evidences the counter-intuitive behaviour of bursty distributions; the more time elapsed since the last event, the more time expected until the next one. We thus see that the network clearly induces burstiness on the activity of nodes.

[1] R. Pastor-Satorras, A. Vázquez, and A. Vespignani, Phys. Rev. Lett. **87**, 258701 (2001).

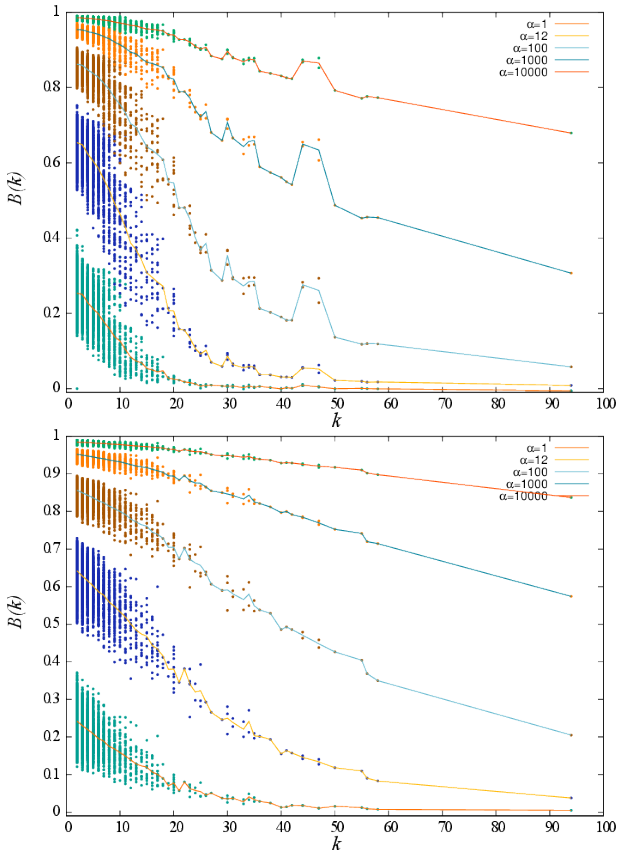

FIG. S3: $B(k)$ for SF graphs with different assortativity r. Top-left: $r = 1.0$. Top-right: $r = 0.5$. Bottom-left: $r = -0.5$. Bottom-right: $r = -1.0$.

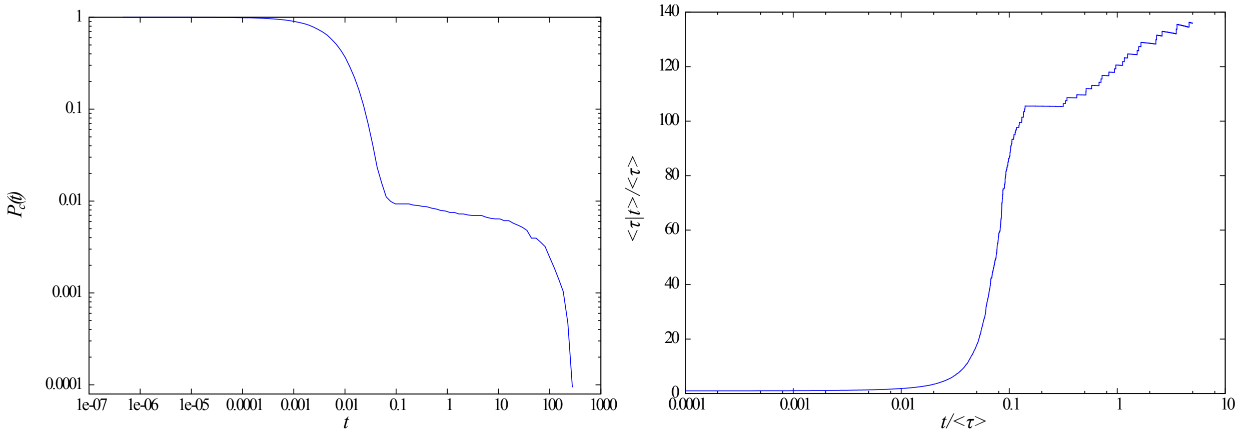

FIG. S4: Left: Cumulative inter-event time distribution function of the node with highest B in the network (for $\alpha = 100$). Right: Normalized conditional average interval $\langle\tau|t\rangle/\langle\tau\rangle$ as a function of $t/\langle\tau\rangle$. We see that it clearly increases above the constant value of 1 expected for a non-bursty Poisson process.
